# Validity and reliability of the Farsi version of the ascertain *dementia* 8-item (AD8-F) informant interview in Iranian patients with mild neurocognitive disorder

**DOI:** 10.1186/s12877-022-03391-y

**Published:** 2022-10-14

**Authors:** Maryam Pourshams, James E. Galvin, Pouya Farokhnezhad Afshar, Pamela Gail Santos, Shahrzad Bazargan-Hejazi, Leila Kamalzadeh, Behnam Shariati, Seyed Kazem Malakouti

**Affiliations:** 1 Department of Psychiatry, Golestan Hospital, Ahvaz Jundishapur University of Medical Sciences, Ahvaz, Iran; 2 Department of Integrated Medical Science, Florida Atlantic University FAU, Florida, USA; 3 Department of Gerontology, School of Behavioral Sciences and Mental Health (Tehran Institute of Psychiatry), Iran University of Medical Sciences, Tehran, Iran; 4 Charles R. Drew University of Medicine and Science, Los Angeles, USA; 5 Department of Psychiatry, Charles R. Drew University of Medicine and Science & David Geffen School of Medicine, Los Angeles, USA; 6 Mental Health Research Center, Iran University of Medical Sciences, Tehran, Iran; 7 Mental Health Research Center, Iran University of Medical Sciences, Tehran, Iran; 8 Behavioral Sciences and Mental Health, Iran University of Medical Sciences, Tehran, Iran

**Keywords:** Aged, Cognition disorders, Cognitive assessment screening instrument

## Abstract

**Background:**

For screening and distinguishing between mild neurocognitive disorder (*mNCD*) and normal cognitive age-related changes in primary care centers, a simple and practical tool is necessary. Therefore, this study aims to determine the validity and reliability of the Farsi version of the Ascertain *Dementia* 8-item (*AD8-F*) informant interview in patients with mNCD.

**Methods:**

This is a study of the psychometric properties of the Farsi AD8. The participants include sixty informant-patient dyads with mNCD and sixty controls with normal cognition. The AD8 was compared to the mini-mental state examination (*MMSE*) and the Mini-Cog. As a gold standard, the Diagnostic and Statistical Manual of Mental Disorders, Fifth Edition (*DSM*-*5*) criteria for mNCD was used. The reliability was measured using internal consistency and test-retest. Validity was assessed by evaluating the content, concurrent, and construct validity. Data were analyzed via Cronbach’s α, Pearson correlation, independent t-test, and analysis of variance (ANOVA) and area under the curve (AUC) by statistical package for the social sciences (*SPSS*) v.23.

**Results:**

Cronbach’s α was 0.71. Test-retest reproducibility was 0.8. The AD8 had inverse correlations with the Mini-Cog (*r =* − 0.70, *P* < 0.01) and MMSE (*r =* − 0.56, *P <* 0.01). The area under the curve was 0.88. The optimal cutoff score was > 2. Sensitivity and specificity were 80 and 83%, respectively. The positive predictive value was 83%. The negative predictive value was 81%.

**Conclusion:**

Our results suggest that this tool can be used as a screening tool to detect a mild neurocognitive disorder in primary care centers.

## Background

Due to increases in life expectancy and greater risk factor burden, the number of people with dementia is rising worldwide, particularly in low- and middle-income countries (LMIC) [1]. Dementia, a chronic, progressive neurocognitive disorder, plays an immense role physically, emotionally, and economically not only in the patient but also in families, caregivers, and communities [2]. Despite its increasing prevalence, only 20-50% of individuals who meet the criteria for dementia are diagnosed by a clinician, and this rate is lower in low- and middle-income countries [3]. Of those diagnosed, many are identified when it is too late to optimize treatment or plan for their future [3]. Therefore, timely detection of neurocognitive disorders is important for better health outcomes.

Various screening tools are available for cognitive impairment, including the Mini-Mental State Examination (MMSE), General Practitioner Assessment of Cognition (GPCOG), Memory Impairment Screen (MIS), and Mini-Cog; however, many are unsuitable for primary care use in LMIC due to lengthy administration and susceptibility to education and cultural bias [4]. Additionally, such tests may be insensitive to the early signs of dementia or influenced by premorbid cognitive conditions, and extensive training about them may be required to administer [5]. To mitigate this gap, in 2005, Galvin et al. developed the Eight-item Informant Interview to Differentiate Aging and Dementia, also known as the Ascertainment Dementia 8-item Questionnaire (AD8) [5]. The AD8 is an informant-based cognitive screening tool consisting of eight yes or no questions based on changes in memory, orientation, judgment, and function [6]. With a cutoff of two or greater predicting dementia, scores range from zero to eight [6]. Since it is sensitive to the earliest signs of cognitive change, requires no formal training to administer, takes only three minutes to complete, and can be administered in various settings such as clinics, home, or over the phone, it is advantageous over other cognitive screening tools. Moreover, it is not affected by the patient’s age, gender, culture, education level, or premorbid evaluation [5]. Consequently, the AD8 has been translated and validated in different languages (i.e., Spanish [7], French [8], Portuguese [9], Norwegian [10], Chinese [11], Korean [12], Indonesian [13], Filipino [14]), Chinese [15, 16], Greek [17, 18], Arabic [19], Turkish [20] and various settings (i.e., primary care, emergency departments, research) across the world [21–24].

The Islamic Republic of Iran is a culturally diverse country located in the Middle East. Its challenged economy places Iran among the LMIC [25]. Much like the rest of the world, its population of almost 83 million is growing old [26, 27]. Life expectancy, which is 74.6 years for women and 72.1 years for men, has seen an upward trend over the years [26], adding to the growing aged population. While comprising 9.6% of the population in 2016, the proportion of Iranians aged 60 and older is expected to increase to 10.5% by 2025 and 21.7% by 2050 [28]. Notably, although Iran’s literacy rate is increasing, the literacy rate of persons aged 65 and over is merely 37% [27].

In congruence with the growing elderly population, the prevalence of dementia in Iran is more likely to increase [29]. While the prevalence of dementia is 7.9%, only about 21% who meet the criteria for dementia are diagnosed [29]. This diagnosis gap deprives many of timely interventions and urges better detection of dementia. Considering the high level of illiteracy in this population, i.e., 63% [27], and various cultures that are practiced, the AD8 as an informant-based assessment of intraindividual change is a suitable screening tool for timely detection of dementia in this country. This study aims to determine the validity and reliability of the Farsi Version of AD8 Informant Interview in Patients with Mild Neurocognitive Disorder.

## Methods

### Translation

Before translating and validating, permission was obtained from the developer of the AD8 at Washington University [30]. The AD8 was translated in three steps using guidelines for cross-cultural adaptation [31, 32]. In the first step, the forward translation, two bilingual native Farsi translators independently translated the original AD8 into Farsi. Both translations were then reviewed by an expert committee consisting of two linguists, a psychologist, and two geriatric psychiatrists. After evaluating for conceptual equivalency and resolving discrepancies, the expert committee synthesized one common translation. In the second step, the back translation, two bilingual native English translators independently translated the common Farsi translation back into English. The expert committee then compared the English back translations with the original version for any inconsistencies. Once discrepancies were resolved, a prefinal translation was drafted. The final step was the pilot study, wherein the prefinal translation was administered to the informants of 20 individuals aged 60 and older. After completion of the questionnaire, the informants were interviewed for probing their understanding of what each item, their corresponding response meant, and if they found any items confusing or difficult to answer. Based on the feedback, the expert committee composed a final Farsi version of the AD8 (AD8-F).

### Study recruitment and participants

From the referrals of a geriatric medicine and memory disorder specialist, the participants were recruited from two outpatient clinics in Tehran, Iran, between January 2020 and July 2021. Eligible participants must fulfill the Diagnostic and Statistical Manual of Mental Disorders, 5th edition (DSM-5) criteria for mild neurocognitive disorder (mNCD) [33]. Additionally, the patient should be at least 60 years of age, completed at least 4 years of formal education, intact vision and hearing, able to communicate verbally, with an available informant were all essential for mNCD patients. Additionally, control subjects should be at least 60 years of age, completed at least 4 years of formal education, hintact vision and hearing, and able to communicate verbally, and with an available informant. The participants with physician-diagnosed depression, schizophrenia, epilepsy, substance abuse, a history of significant head injury, or any disorders influencing task performances possibly, were excluded from this study. Cognitively normal control subjects were recruited by referrals from caregivers and relatives of mNCD patients to compare cognitive screening tests. Control subjects were evaluated by two geriatric psychiatrists to rule out cognitive impairment. Additionally, control subjects had to be at least 60 years old, had completed at least 4 years of formal education, had intact vision and hearing abilities, were capable of verbal communication, and had an available informant. We selected 60 participants for each group through convenience sampling. The sample size was determined based on Cronbach’s alpha estimation (expected Cronbach’s alpha: 0.8, α: 0.05, β: 0.2, number of items: 8) [34, 35]. Before conducting this study, approval was obtained from the ethics committee at the Iran University of Medical Sciences; and this study has been performed in accordance with the Declaration of Helsinki; all participants and their informants gave their written informed consent.

### Psychometric evaluation

The AD8-F was administered to the informants of 60 patients with mNCD and 60 controls. To investigate test-retest reliability, participants were asked to return two weeks after the initial examination. A total of 30 participants from each group returned to complete the retest. The facets of validity tested were content validity, concurrent validity, and construct validity. Content validity was evaluated by an expert committee during the translation process. The construct and concurrent validities were assessed by correlating and comparing the AD8-F with the Mini-Mental State Exam (MMSE) and the Mini-Cog. The MMSE is a very widely used and studied cognitive impairment screening tool assessing orientation, memory, concentration, language, and praxis [36–38]. A Farsi version showed reliability and validity, with a cut-off score of 23 (out of 30) or below suggestive of dementia [39]. The Mini-Cog is a brief cognitive test assessing cognitive function, memory, language comprehension, visual-motor skills, and executive function [40]. A Farsi version showed reliability and validity, with a cutoff score of two (out of five) or below suggestive of dementia [41]. The DSM-5 criteria for minor neurocognitive disorder were used as the gold standard for detecting mild cognitive impairment by two geriatric psychiatrists. The Persian version of all cognitive screening tools used in this study was administered by two geriatric psychiatrists who were not blinded to the results of the tests.

### Data analysis

All data were analyzed using the Statistical Package for the Social Sciences software (SPSS 23). The Chi-square test was used to obtain differences in demographic characteristics that were categorical, while one-way ANOVA was used to compare mean differences. Cronbach’s alpha (α) was used to report internal consistency [42]. The Pearson’s correlation coefficient (Pearson’s r) was used to assess test-retest reliability and determine the correlation between the AD8, MMSE, and Mini-Cog scores. Alpha values ≥0.70 were considered an acceptable threshold for reliability. Correlations of 0 to ±0.3, ±0.3 to ±0.5, ±0.5 to ±0.7, ±0.7 to ±0.9, and ± 0.9 to ±1.0 were interpreted as negligible, low, strong, high, or very high, respectively [43]. Receiver Operating Characteristic (ROC) curve and area under the curve (AUC) analyses were used to determine diagnostic accuracy, optimal cut-off scores for sensitivity and specificity, and positive and negative predictive values (PPV and NPV, respectively).

## Results

A Farsi adaptation of the AD8 was created after a rigorous translation process. An expert committee evaluated its content for relevance, representativeness, and technical quality to establish content validity following the best practices for developing and validating scales [42].

As indicated in Table 1, no statistically significant differences existed in the demographic characteristics of the mNCD and control groups. The average age of participants was 69 years old. There were more women in the mNCD group compared to the control group (60% versus 43%). During the assessment, 50 and 53% of the participants in the mNCD and control groups, respectively, were married, and 62 and 70% of the participants in the mNCD and control groups, respectively. Most completed only primary level education. There were more homemakers in the mNCD group compared to the control group (53% versus 37%).

**Table 1:** Demographic characteristics of the participants

	mNCD (*n =* 60)	Control (*n =* 60)	*P*-value
Age, mean ± SD, y	68.8 ± 8.0	69.4 ± 7.4	0.67
Sex, n (%)			0.7
Female	36 (60)	26 (43)	
Male	24 (40)	34 (57)	
Marital status, n (%)			0.8
Married	30 (50)	32 (53)	
Widowed	21 (35)	18 (30)	
Single	9 (15)	10 (17)	
Education level, n (%)			0.31
Primary school	37 (62)	42 (70)	
Secondary school	19 (32)	12 (20)	
College or higher	4 (7)	6 (10)	
Employment status, n (%)			0.2
Homemaker	32 (53)	22 (37)	
Retired	12 (20)	20 (33)	
Employed	9 (15)	12 (20)	
Unemployed	7 (12)	6 (10)	

### Reliability and validity analyses

Test-retest reliability (*r =* 0.8) and internal consistency (Cronbach’s α = 0.71) were acceptable. Concerning concurrent (criterion) validity, as illustrated in Table 2, statistically higher AD8-F scores and lower MMSE and Mini-Cog scores were observed in the mNCD compared to the control.

**Table 2:** Mean and standard deviation of AD8, MMSE, and *Mini-Cog*

	mNCD mean ± SD	Control mean ± SD	*T*-value	*P*-value
AD8-F (0-8)^a^	3.8 **±** 1.8	1.2 **±** 1.2	9.4	0.0001
MMSE (0-30)^b^	28.4 **±** 0.8	29.8 **±** 0.5	11.97	0.0001
Mini-Cog (0-5)^b^	1.8 **±** 1.4	4.5 **±** 0.8	13.03	0.0001

*mNCD *Mild Neurocognitive Disorder,* MMSE *Mini-Mental State Exam

^a^*Higher scores equal greater cognitive impairment. bLower scores equal greater cognitive impairment*

To establish the construct validity, the association between AD8-F, Mini-Cog, and MMSE scores was calculated (Table 3). The AD8-F scores were highly and strongly negatively correlated with the Mini-Cog and MMSE scores, respectively.

**Table 3:** Correlation between AD8 scores and other cognitive screening tools

	r	*P*-value
Mini-Cog	−0.70	< 0.01
MMSE	−0.56	< 0.01

*MMSE* Mini-Mental State Exam

**Fig. 1:**
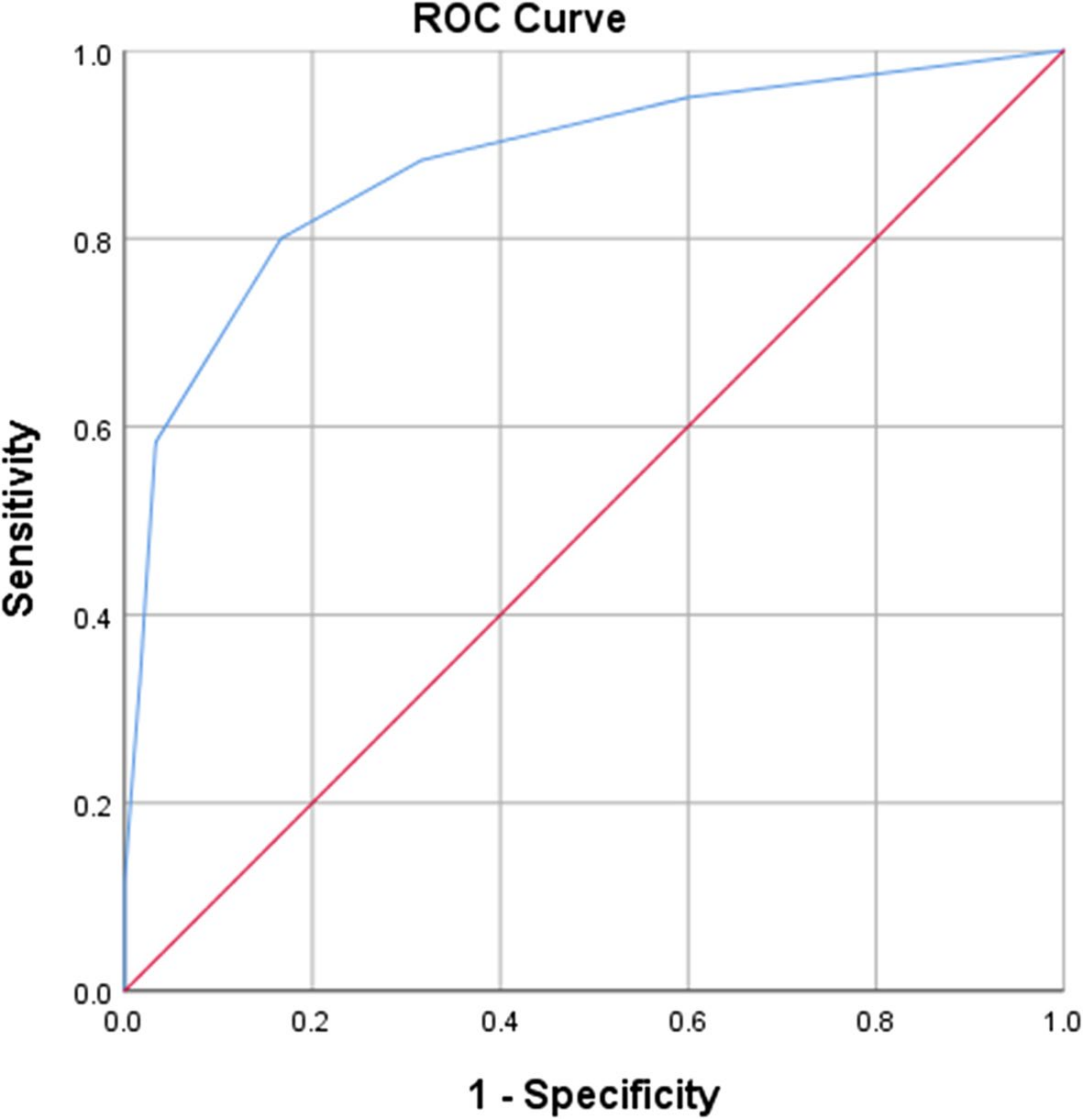
Receiver operating characteristic (ROC curve) for AD8-F; area under the curve: 0.88

The AUC was 88% (95% confidence interval, 0.82-0.94) (Fig. 1), suggesting an excellent ability to discriminate between mNCD and normal cognition [44]. Sensitivity (83%) and specificity (80%) yielded optimal results at a cut-off score of 2. The PPV was 83%, which refers to the likelihood that someone with an AD score of > 2 has dementia. NPV was 81%, which refers to the likelihood that someone with an AD score of ≤2 has no dementia (Table 4).

**Table 4:** Measures of discrimination for diagnosis of mNCD versus normal cognition

	AD8	MMSE	Mini-Cog
AUC (95% CI)	0.88 (0.82 - 0.94)	0.93 (0.89 - 0.98)	0.94 (0.90 – 0.98)
Cut-off score	> 2	< 27	< 2
Sensitivity	0.80	0.81	0.98
Specificity	0.83	1.00	0.77
PPV	0.83	1.00	0.98
NPV	0.81	0.53	0.81

*AUC* Area under the curve, CI Confidence intervals 95%.

*PPV* Positive Predictive Value, *NPV* Negative Predictive Value.

## Discussion

Translating and assessing the AD8’s psychometric properties for use in the Iranian elderlies were the aims of this study. The AD8-F took less than 3 minutes to complete. Test-retest reliability was acceptable (*r =* 0.8), indicating consistency of respondents’ scores over time. Instead of Pearson’s r, previous studies used intraclass correlation coefficient (ICC ≥ 0.80) or weighted kappa (weighted k ≥ 0.80) to establish test-retest reliability [6, 9, 11, 12, 17]. Cronbach’s alpha (α = 0.71) indicated that all items of the questionnaire measured the same concept.

Similar to previous studies, the current study observed a negative correlation between the Farsi versions of AD8 and MMSE and the Mini-Cog [8, 11, 15, 17, 18, 23]. This study used the DSM-5 criteria for the minor neurocognitive disorder. The DSM-5 is a universally accepted and reliable method of diagnosing neurocognitive disorders. The AD8-F had excellent discriminatory power in detecting mNCD [AUC 0.88 (0.82- 0.94), cut-off: > 2, sensitivity: 0.80, specificity: 0.83, PPV: 0.83 and NPV: 0.8. So, the AD8-F could discriminate those with mild neurocognitive disorder (diagnosed in terms of the Diagnostic and Statistical Manual of Mental Disorders, 5th edition), from individuals who had normal cognition. Previous studies of the AD8 reported a cut-off score of two to three or greater, with a sensitivity and specificity of 0.68 to 0.97 and 0.61 to 0.93, respectively [5, 7, 9, 15, 19, 20, 22, 23].

## Conclusion

The Farsi adaptation of the AD8 retained the psychometric properties of the original English version and therefore is a reliable and valid screening tool for detecting mNCD in the Iranian elderly population. Mild neurocognitive disorder, known as mild cognitive impairment or MCI, is the prodromal of major neurocognitive disorder (also known as dementia). To treat reversible causes of mNCD, like medication side effects and metabolic derangements, early detection of cognitive impairment is essential. While there is no proven treatment for non-reversible causes of dementia, disease-modifying therapies and interventions have been shown to delay disease progression to overt dementia and are more effective if administered earlier in the disease course. The AD8-F, therefore, has the potential to detect the earliest signs of cognitive impairment, thereby improving the health care outcomes of patients and their families.

## Data Availability

All data generated or analyzed during this study are included in this published article [and its supplementary information files].

## Abbreviations

*Mncd*: Mild neurocognitive disorder
*AD8*: Ascertain *Dementia* 8-item
*MMSE*: Mini-Mental State examination
*DSM*-*5*: Diagnostic and Statistical Manual of Mental Disorders, Fifth Edition
*ANOVA*: Analysis of variance
*AUC*: Area under the curve
*SPSS*: Statistical package for the social sciences
*LMIC*: Low- and middle-income countries
*GPCOG*: General Practitioner Assessment of Cognition
*MIS*: Memory Impairment Screen
*PPV*: Positive predictive values
*NPV*: Negative predictive values
*ICC*: Intraclass correlation coefficient
